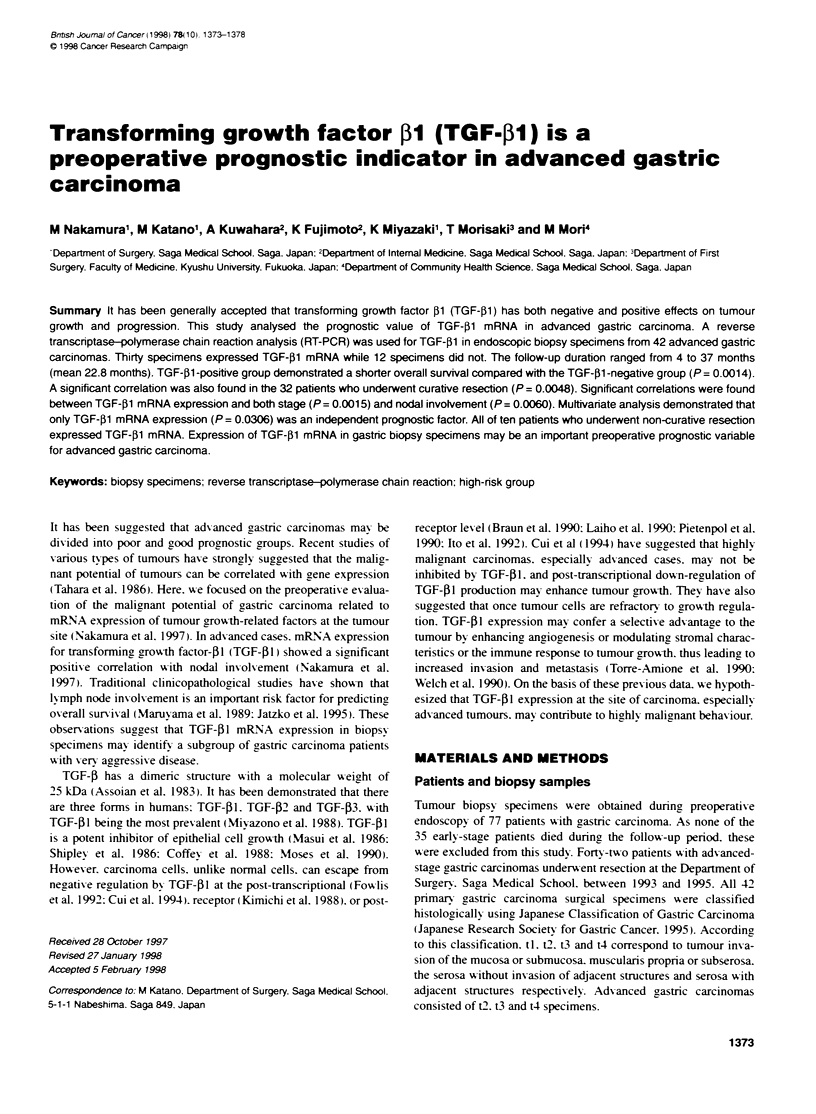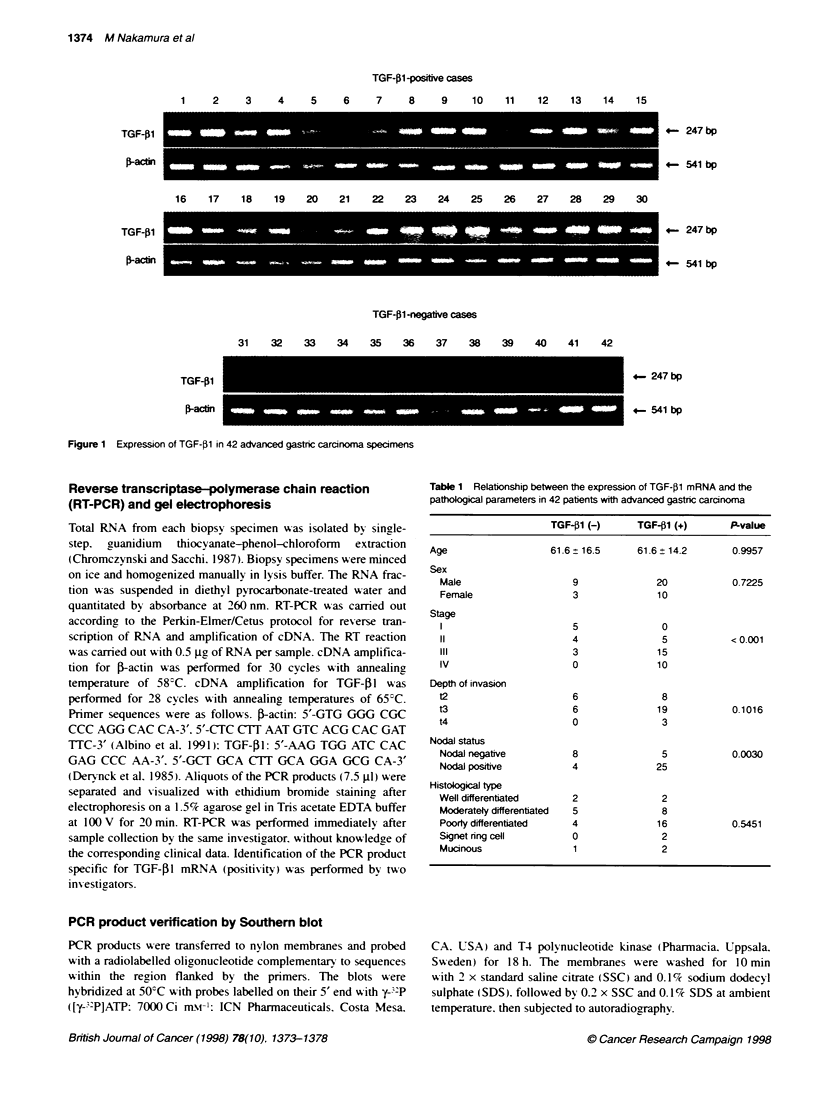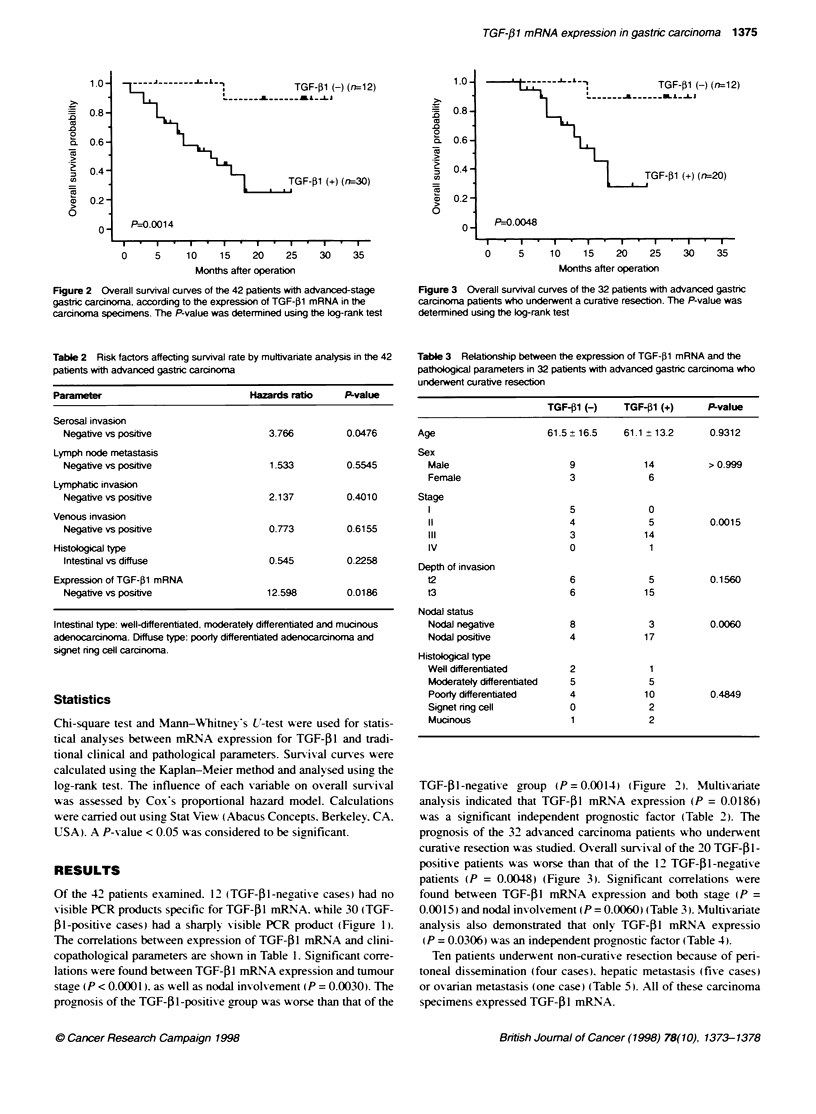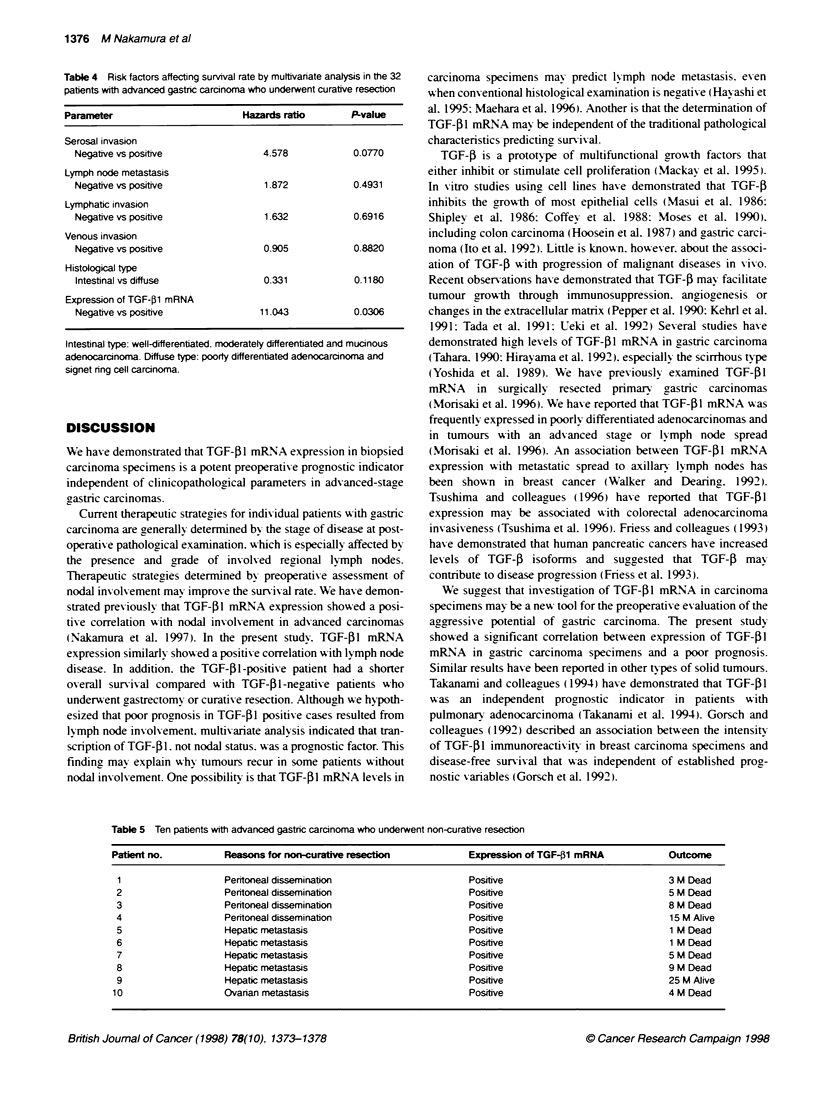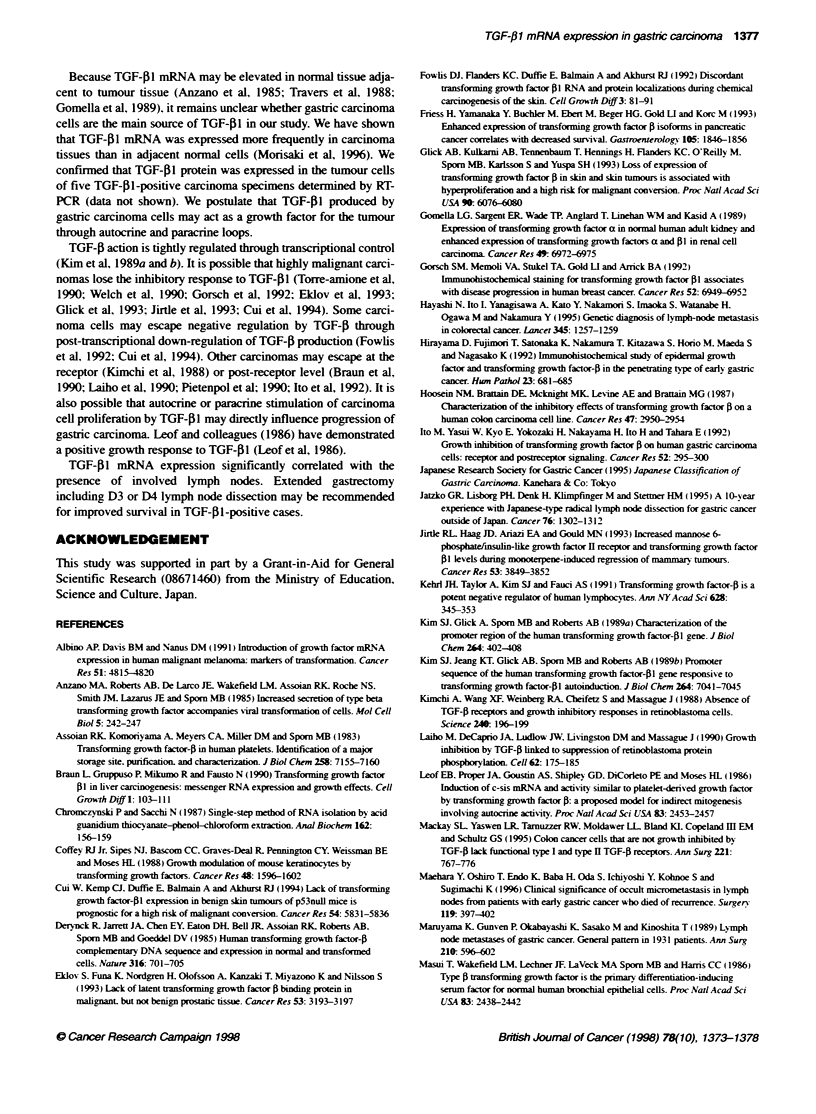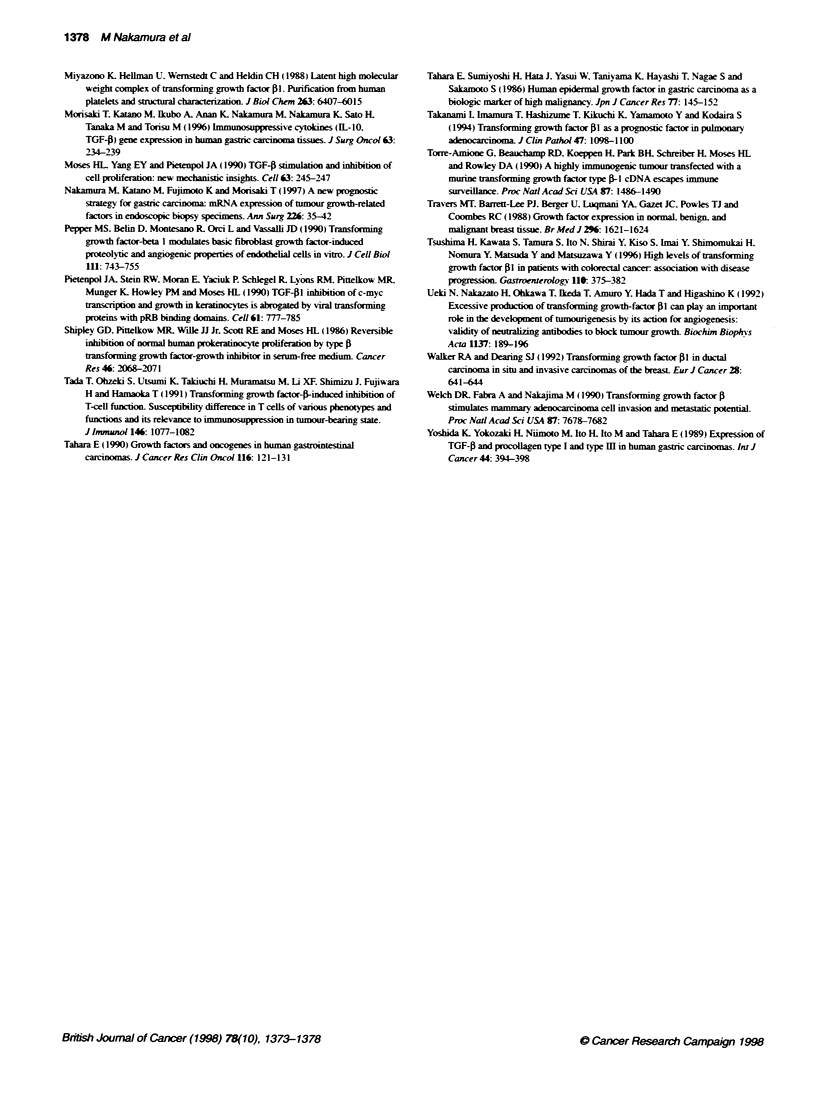# Transforming growth factor beta1 (TGF-beta1) is a preoperative prognostic indicator in advanced gastric carcinoma.

**DOI:** 10.1038/bjc.1998.687

**Published:** 1998-11

**Authors:** M. Nakamura, M. Katano, A. Kuwahara, K. Fujimoto, K. Miyazaki, T. Morisaki, M. Mori

**Affiliations:** Department of Surgery, Saga Medical School, Japan.

## Abstract

**Images:**


British Journal of Cancer (1998) 78(10), 1373–1378
© 1998 Cancer Research Campaign

### Transforming growth factor β1 (TGF-β1) is a preoperative prognostic indicator in advanced gastric carcinoma

**M Nakamura[1], M Katano[1], A Kuwahara[2], K Fujimoto[2], K Miyazaki[1], T Morisaki[3] and M Mori[4]**

[1]Department of Surgery, Saga Medical School, Saga, Japan; [2]Department of Internal Medicine, Saga Medical School, Saga, Japan; [3]Department of First Surgery, Faculty of Medicine, Kyushu University, Fukuoka, Japan; [4]Department of Community Health Science, Saga Medical School, Saga, Japan

**Summary** It has been generally accepted that transforming growth factor β1 (TGF-β1) has both negative and positive effects on tumour growth and progression. This study analysed the prognostic value of TGF-β1 mRNA in advanced gastric carcinoma. A reverse transcriptase–polymerase chain reaction analysis (RT-PCR) was used for TGF-β1 in endoscopic biopsy specimens from 42 advanced gastric carcinomas. Thirty specimens expressed TGF-β1 mRNA while 12 specimens did not. The follow-up duration ranged from 4 to 37 months (mean 22.8 months). TGF-β1-positive group demonstrated a shorter overall survival compared with the TGF-β1-negative group ($P = 0.0014$). A significant correlation was also found in the 32 patients who underwent curative resection ($P = 0.0048$). Significant correlations were found between TGF-β1 mRNA expression and both stage ($P = 0.0015$) and nodal involvement ($P = 0.0060$). Multivariate analysis demonstrated that only TGF-β1 mRNA expression ($P = 0.0306$) was an independent prognostic factor. All of ten patients who underwent non-curative resection expressed TGF-β1 mRNA. Expression of TGF-β1 mRNA in gastric biopsy specimens may be an important preoperative prognostic variable for advanced gastric carcinoma.

**Keywords:** biopsy specimens; reverse transcriptase–polymerase chain reaction; high-risk group

It has been suggested that advanced gastric carcinomas may be divided into poor and good prognostic groups. Recent studies of various types of tumours have strongly suggested that the malignant potential of tumours can be correlated with gene expression (Tahara et al, 1986). Here, we focused on the preoperative evaluation of the malignant potential of gastric carcinoma related to mRNA expression of tumour growth-related factors at the tumour site (Nakamura et al, 1997). In advanced cases, mRNA expression for transforming growth factor-β1 (TGF-β1) showed a significant positive correlation with nodal involvement (Nakamura et al, 1997). Traditional clinicopathological studies have shown that lymph node involvement is an important risk factor for predicting overall survival (Maruyama et al, 1989; Jatzko et al, 1995). These observations suggest that TGF-β1 mRNA expression in biopsy specimens may identify a subgroup of gastric carcinoma patients with very aggressive disease.

TGF-β has a dimeric structure with a molecular weight of 25 kDa (Assoian et al, 1983). It has been demonstrated that there are three forms in humans: TGF-β1, TGF-β2 and TGF-β3, with TGF-β1 being the most prevalent (Miyazono et al, 1988). TGF-β1 is a potent inhibitor of epithelial cell growth (Masui et al, 1986; Shipley et al, 1986; Coffey et al, 1988; Moses et al, 1990). However, carcinoma cells, unlike normal cells, can escape from negative regulation by TGF-β1 at the post-transcriptional (Fowlis et al, 1992; Cui et al, 1994), receptor (Kimichi et al, 1988), or post-receptor level (Braun et al, 1990; Laiho et al, 1990; Pietenpol et al, 1990; Ito et al, 1992). Cui et al (1994) have suggested that highly malignant carcinomas, especially advanced cases, may not be inhibited by TGF-β1, and post-transcriptional down-regulation of TGF-β1 production may enhance tumour growth. They have also suggested that once tumour cells are refractory to growth regulation, TGF-β1 expression may confer a selective advantage to the tumour by enhancing angiogenesis or modulating stromal characteristics or the immune response to tumour growth, thus leading to increased invasion and metastasis (Torre-Amione et al, 1990; Welch et al, 1990). On the basis of these previous data, we hypothesized that TGF-β1 expression at the site of carcinoma, especially advanced tumours, may contribute to highly malignant behaviour.

*Received 28 October 1997*
*Revised 27 January 1998*
*Accepted 5 February 1998*

*Correspondence to:* M Katano, Department of Surgery, Saga Medical School, 5-1-1 Nabeshima, Saga 849, Japan

### MATERIALS AND METHODS

### Patients and biopsy samples

Tumour biopsy specimens were obtained during preoperative endoscopy of 77 patients with gastric carcinoma. As none of the 35 early-stage patients died during the follow-up period, these were excluded from this study. Forty-two patients with advanced-stage gastric carcinomas underwent resection at the Department of Surgery, Saga Medical School, between 1993 and 1995. All 42 primary gastric carcinoma surgical specimens were classified histologically using Japanese Classification of Gastric Carcinoma (Japanese Research Society for Gastric Cancer, 1995). According to this classification, t1, t2, t3 and t4 correspond to tumour invasion of the mucosa or submucosa, muscularis propria or subserosa, the serosa without invasion of adjacent structures and serosa with adjacent structures respectively. Advanced gastric carcinomas consisted of t2, t3 and t4 specimens.

**Figure 1** Expression of TGF-β1 in 42 advanced gastric carcinoma specimens

### Reverse transcriptase–polymerase chain reaction (RT-PCR) and gel electrophoresis

Total RNA from each biopsy specimen was isolated by single-step, guanidium thiocyanate–phenol–chloroform extraction (Chromczynski and Sacchi, 1987). Biopsy specimens were minced on ice and homogenized manually in lysis buffer. The RNA fraction was suspended in diethyl pyrocarbonate-treated water and quantitated by absorbance at 260 nm. RT-PCR was carried out according to the Perkin-Elmer/Cetus protocol for reverse transcription of RNA and amplification of cDNA. The RT reaction was carried out with 0.5 μg of RNA per sample. cDNA amplification for β-actin was performed for 30 cycles with annealing temperature of 58°C. cDNA amplification for TGF-β1 was performed for 28 cycles with annealing temperatures of 65°C. Primer sequences were as follows. β-actin: 5′-GTG GGG CGC CCC AGG CAC CA-3′, 5′-CTC CTT AAT GTC ACG CAC GAT TTC-3′ (Albino et al, 1991); TGF-β1: 5′-AAG TGG ATC CAC GAG CCC AA-3′, 5′-GCT GCA CTT GCA GGA GCG CA-3′ (Derynck et al, 1985). Aliquots of the PCR products (7.5 μl) were separated and visualized with ethidium bromide staining after electrophoresis on a 1.5% agarose gel in Tris acetate EDTA buffer at 100 V for 20 min. RT-PCR was performed immediately after sample collection by the same investigator, without knowledge of the corresponding clinical data. Identification of the PCR product specific for TGF-β1 mRNA (positivity) was performed by two investigators.

**Table 1** Relationship between the expression of TGF-β1 mRNA and the pathological parameters in 42 patients with advanced gastric carcinoma

| | TGF-β1 (–) | TGF-β1 (+) | *P*-value |
|---|---|---|---|
| Age | 61.6 ± 16.5 | 61.6 ± 14.2 | 0.9957 |
| Sex | | | |
| Male | 9 | 20 | 0.7225 |
| Female | 3 | 10 | |
| Stage | | | |
| I | 5 | 0 | |
| II | 4 | 5 | < 0.001 |
| III | 3 | 15 | |
| IV | 0 | 10 | |
| Depth of invasion | | | |
| t2 | 6 | 8 | |
| t3 | 6 | 19 | 0.1016 |
| t4 | 0 | 3 | |
| Nodal status | | | |
| Nodal negative | 8 | 5 | 0.0030 |
| Nodal positive | 4 | 25 | |
| Histological type | | | |
| Well differentiated | 2 | 2 | |
| Moderately differentiated | 5 | 8 | |
| Poorly differentiated | 4 | 16 | 0.5451 |
| Signet ring cell | 0 | 2 | |
| Mucinous | 1 | 2 | |

### PCR product verification by Southern blot

PCR products were transferred to nylon membranes and probed with a radiolabelled oligonucleotide complementary to sequences within the region flanked by the primers. The blots were hybridized at 50°C with probes labelled on their 5′ end with γ-$^{32}$P ([γ-$^{32}$P]ATP: 7000 Ci mM$^{-1}$; ICN Pharmaceuticals, Costa Mesa, CA, USA) and T4 polynucleotide kinase (Pharmacia, Uppsala, Sweden) for 18 h. The membranes were washed for 10 min with 2 × standard saline citrate (SSC) and 0.1% sodium dodecyl sulphate (SDS), followed by 0.2 × SSC and 0.1% SDS at ambient temperature, then subjected to autoradiography.

*© Cancer Research Campaign 1998*

**Figure 2** Overall survival curves of the 42 patients with advanced-stage gastric carcinoma, according to the expression of TGF-β1 mRNA in the carcinoma specimens. The P-value was determined using the log-rank test

**Figure 3** Overall survival curves of the 32 patients with advanced gastric carcinoma patients who underwent a curative resection. The P-value was determined using the log-rank test

**Table 2** Risk factors affecting survival rate by multivariate analysis in the 42 patients with advanced gastric carcinoma

| Parameter | Hazards ratio | P-value |
|---|---|---|
| Serosal invasion | | |
| Negative vs positive | 3.766 | 0.0476 |
| Lymph node metastasis | | |
| Negative vs positive | 1.533 | 0.5545 |
| Lymphatic invasion | | |
| Negative vs positive | 2.137 | 0.4010 |
| Venous invasion | | |
| Negative vs positive | 0.773 | 0.6155 |
| Histological type | | |
| Intestinal vs diffuse | 0.545 | 0.2258 |
| Expression of TGF-β1 mRNA | | |
| Negative vs positive | 12.598 | 0.0186 |

Intestinal type: well-differentiated, moderately differentiated and mucinous adenocarcinoma. Diffuse type: poorly differentiated adenocarcinoma and signet ring cell carcinoma.

### Statistics

Chi-square test and Mann–Whitney's U-test were used for statistical analyses between mRNA expression for TGF-β1 and traditional clinical and pathological parameters. Survival curves were calculated using the Kaplan–Meier method and analysed using the log-rank test. The influence of each variable on overall survival was assessed by Cox's proportional hazard model. Calculations were carried out using Stat View (Abacus Concepts, Berkeley, CA, USA). A P-value < 0.05 was considered to be significant.

### RESULTS

Of the 42 patients examined, 12 (TGF-β1-negative cases) had no visible PCR products specific for TGF-β1 mRNA, while 30 (TGF-β1-positive cases) had a sharply visible PCR product (Figure 1). The correlations between expression of TGF-β1 mRNA and clinicopathological parameters are shown in Table 1. Significant correlations were found between TGF-β1 mRNA expression and tumour stage (P < 0.0001), as well as nodal involvement (P = 0.0030). The prognosis of the TGF-β1-positive group was worse than that of the TGF-β1-negative group (P = 0.0014) (Figure 2). Multivariate analysis indicated that TGF-β1 mRNA expression (P = 0.0186) was a significant independent prognostic factor (Table 2). The prognosis of the 32 advanced carcinoma patients who underwent curative resection was studied. Overall survival of the 20 TGF-β1-positive patients was worse than that of the 12 TGF-β1-negative patients (P = 0.0048) (Figure 3). Significant correlations were found between TGF-β1 mRNA expression and both stage (P = 0.0015) and nodal involvement (P = 0.0060) (Table 3). Multivariate analysis also demonstrated that only TGF-β1 mRNA expressio (P = 0.0306) was an independent prognostic factor (Table 4).

**Table 3** Relationship between the expression of TGF-β1 mRNA and the pathological parameters in 32 patients with advanced gastric carcinoma who underwent curative resection

| | TGF-β1 (–) | TGF-β1 (+) | P-value |
|---|---|---|---|
| Age | 61.5 ± 16.5 | 61.1 ± 13.2 | 0.9312 |
| Sex | | | |
| Male | 9 | 14 | > 0.999 |
| Female | 3 | 6 | |
| Stage | | | |
| I | 5 | 0 | |
| II | 4 | 5 | 0.0015 |
| III | 3 | 14 | |
| IV | 0 | 1 | |
| Depth of invasion | | | |
| t2 | 6 | 5 | 0.1560 |
| t3 | 6 | 15 | |
| Nodal status | | | |
| Nodal negative | 8 | 3 | 0.0060 |
| Nodal positive | 4 | 17 | |
| Histological type | | | |
| Well differentiated | 2 | 1 | |
| Moderately differentiated | 5 | 5 | |
| Poorly differentiated | 4 | 10 | 0.4849 |
| Signet ring cell | 0 | 2 | |
| Mucinous | 1 | 2 | |

Ten patients underwent non-curative resection because of peritoneal dissemination (four cases), hepatic metastasis (five cases) or ovarian metastasis (one case) (Table 5). All of these carcinoma specimens expressed TGF-β1 mRNA.

Table 4 Risk factors affecting survival rate by multivariate analysis in the 32 patients with advanced gastric carcinoma who underwent curative resection

| Parameter | Hazards ratio | P-value |
|---|---|---|
| Serosal invasion | | |
| Negative vs positive | 4.578 | 0.0770 |
| Lymph node metastasis | | |
| Negative vs positive | 1.872 | 0.4931 |
| Lymphatic invasion | | |
| Negative vs positive | 1.632 | 0.6916 |
| Venous invasion | | |
| Negative vs positive | 0.905 | 0.8820 |
| Histological type | | |
| Intestinal vs diffuse | 0.331 | 0.1180 |
| Expression of TGF-β1 mRNA | | |
| Negative vs positive | 11.043 | 0.0306 |

Intestinal type: well-differentiated, moderately differentiated and mucinous adenocarcinoma. Diffuse type: poorly differentiated adenocarcinoma and signet ring cell carcinoma.

### DISCUSSION

We have demonstrated that TGF-β1 mRNA expression in biopsied carcinoma specimens is a potent preoperative prognostic indicator independent of clinicopathological parameters in advanced-stage gastric carcinomas.

Current therapeutic strategies for individual patients with gastric carcinoma are generally determined by the stage of disease at post-operative pathological examination, which is especially affected by the presence and grade of involved regional lymph nodes. Therapeutic strategies determined by preoperative assessment of nodal involvement may improve the survival rate. We have demonstrated previously that TGF-β1 mRNA expression showed a positive correlation with nodal involvement in advanced carcinomas (Nakamura et al. 1997). In the present study, TGF-β1 mRNA expression similarly showed a positive correlation with lymph node disease. In addition, the TGF-β1-positive patient had a shorter overall survival compared with TGF-β1-negative patients who underwent gastrectomy or curative resection. Although we hypothesized that poor prognosis in TGF-β1 positive cases resulted from lymph node involvement, multivariate analysis indicated that transcription of TGF-β1, not nodal status, was a prognostic factor. This finding may explain why tumours recur in some patients without nodal involvement. One possibility is that TGF-β1 mRNA levels in carcinoma specimens may predict lymph node metastasis, even when conventional histological examination is negative (Hayashi et al. 1995; Maehara et al. 1996). Another is that the determination of TGF-β1 mRNA may be independent of the traditional pathological characteristics predicting survival.

TGF-β is a prototype of multifunctional growth factors that either inhibit or stimulate cell proliferation (Mackay et al. 1995). In vitro studies using cell lines have demonstrated that TGF-β inhibits the growth of most epithelial cells (Masui et al. 1986; Shipley et al. 1986; Coffey et al. 1988; Moses et al. 1990), including colon carcinoma (Hoosein et al. 1987) and gastric carcinoma (Ito et al. 1992). Little is known, however, about the association of TGF-β with progression of malignant diseases in vivo. Recent observations have demonstrated that TGF-β may facilitate tumour growth through immunosuppression, angiogenesis or changes in the extracellular matrix (Pepper et al. 1990; Kehrl et al. 1991; Tada et al. 1991; Ueki et al. 1992) Several studies have demonstrated high levels of TGF-β1 mRNA in gastric carcinoma (Tahara, 1990; Hirayama et al. 1992), especially the scirrhous type (Yoshida et al. 1989). We have previously examined TGF-β1 mRNA in surgically resected primary gastric carcinomas (Morisaki et al. 1996). We have reported that TGF-β1 mRNA was frequently expressed in poorly differentiated adenocarcinomas and in tumours with an advanced stage or lymph node spread (Morisaki et al. 1996). An association between TGF-β1 mRNA expression with metastatic spread to axillary lymph nodes has been shown in breast cancer (Walker and Dearing, 1992). Tsushima and colleagues (1996) have reported that TGF-β1 expression may be associated with colorectal adenocarcinoma invasiveness (Tsushima et al. 1996). Friess and colleagues (1993) have demonstrated that human pancreatic cancers have increased levels of TGF-β isoforms and suggested that TGF-β may contribute to disease progression (Friess et al. 1993).

We suggest that investigation of TGF-β1 mRNA in carcinoma specimens may be a new tool for the preoperative evaluation of the aggressive potential of gastric carcinoma. The present study showed a significant correlation between expression of TGF-β1 mRNA in gastric carcinoma specimens and a poor prognosis. Similar results have been reported in other types of solid tumours. Takanami and colleagues (1994) have demonstrated that TGF-β1 was an independent prognostic indicator in patients with pulmonary adenocarcinoma (Takanami et al. 1994). Gorsch and colleagues (1992) described an association between the intensity of TGF-β1 immunoreactivity in breast carcinoma specimens and disease-free survival that was independent of established prognostic variables (Gorsch et al. 1992).

Table 5 Ten patients with advanced gastric carcinoma who underwent non-curative resection

| Patient no. | Reasons for non-curative resection | Expression of TGF-β1 mRNA | Outcome |
|---|---|---|---|
| 1 | Peritoneal dissemination | Positive | 3 M Dead |
| 2 | Peritoneal dissemination | Positive | 5 M Dead |
| 3 | Peritoneal dissemination | Positive | 8 M Dead |
| 4 | Peritoneal dissemination | Positive | 15 M Alive |
| 5 | Hepatic metastasis | Positive | 1 M Dead |
| 6 | Hepatic metastasis | Positive | 1 M Dead |
| 7 | Hepatic metastasis | Positive | 5 M Dead |
| 8 | Hepatic metastasis | Positive | 9 M Dead |
| 9 | Hepatic metastasis | Positive | 25 M Alive |
| 10 | Ovarian metastasis | Positive | 4 M Dead |

© Cancer Research Campaign 1998

Because TGF-β1 mRNA may be elevated in normal tissue adjacent to tumour tissue (Anzano et al, 1985; Travers et al, 1988; Gomella et al, 1989), it remains unclear whether gastric carcinoma cells are the main source of TGF-β1 in our study. We have shown that TGF-β1 mRNA was expressed more frequently in carcinoma tissues than in adjacent normal cells (Morisaki et al, 1996). We confirmed that TGF-β1 protein was expressed in the tumour cells of five TGF-β1-positive carcinoma specimens determined by RT-PCR (data not shown). We postulate that TGF-β1 produced by gastric carcinoma cells may act as a growth factor for the tumour through autocrine and paracrine loops.

TGF-β action is tightly regulated through transcriptional control (Kim et al, 1989*a* and *b*). It is possible that highly malignant carcinomas lose the inhibitory response to TGF-β1 (Torre-amione et al, 1990; Welch et al, 1990; Gorsch et al, 1992; Eklov et al, 1993; Glick et al, 1993; Jirtle et al, 1993; Cui et al, 1994). Some carcinoma cells may escape negative regulation by TGF-β through post-transcriptional down-regulation of TGF-β production (Fowlis et al, 1992; Cui et al, 1994). Other carcinomas may escape at the receptor (Kimchi et al, 1988) or post-receptor level (Braun et al, 1990; Laiho et al, 1990; Pietenpol et al; 1990; Ito et al, 1992). It is also possible that autocrine or paracrine stimulation of carcinoma cell proliferation by TGF-β1 may directly influence progression of gastric carcinoma. Leof and colleagues (1986) have demonstrated a positive growth response to TGF-β1 (Leof et al, 1986).

TGF-β1 mRNA expression significantly correlated with the presence of involved lymph nodes. Extended gastrectomy including D3 or D4 lymph node dissection may be recommended for improved survival in TGF-β1-positive cases.

### ACKNOWLEDGEMENT

This study was supported in part by a Grant-in-Aid for General Scientific Research (08671460) from the Ministry of Education, Science and Culture, Japan.

### REFERENCES

Albino AP, Davis BM and Nanus DM (1991) Introduction of growth factor mRNA expression in human malignant melanoma: markers of transformation. *Cancer Res* **51**: 4815–4820

Anzano MA, Roberts AB, De Larco JE, Wakefield LM, Assoian RK, Roche NS, Smith JM, Lazarus JE and Sporn MB (1985) Increased secretion of type beta transforming growth factor accompanies viral transformation of cells. *Mol Cell Biol* **5**: 242–247

Assoian RK, Komoriyama A, Meyers CA, Miller DM and Sporn MB (1983) Transforming growth factor-β in human platelets. Identification of a major storage site, purification, and characterization. *J Biol Chem* **258**: 7155–7160

Braun L, Gruppuso P, Mikumo R and Fausto N (1990) Transforming growth factor β1 in liver carcinogenesis: messenger RNA expression and growth effects. *Cell Growth Diff* **1**: 103–111

Chromczynski P and Sacchi N (1987) Single-step method of RNA isolation by acid guanidium thiocyanate–phenol–chloroform extraction. *Anal Biochem* **162**: 156–159

Coffey RJ Jr, Sipes NJ, Bascom CC, Graves-Deal R, Pennington CY, Weissman BE and Moses HL (1988) Growth modulation of mouse keratinocytes by transforming growth factors. *Cancer Res* **48**: 1596–1602

Cui W, Kemp CJ, Duffie E, Balmain A and Akhurst RJ (1994) Lack of transforming growth factor-β1 expression in benign skin tumours of p53null mice is prognostic for a high risk of malignant conversion. *Cancer Res* **54**: 5831–5836

Derynck R, Jarrett JA, Chen EY, Eaton DH, Bell JR, Assoian RK, Roberts AB, Sporn MB and Goeddel DV (1985) Human transforming growth factor-β complementary DNA sequence and expression in normal and transformed cells. *Nature* **316**: 701–705

Eklov S, Funa K, Nordgren H, Olofsson A, Kanzaki T, Miyazono K and Nilsson S (1993) Lack of latent transforming growth factor β binding protein in malignant, but not benign prostatic tissue. *Cancer Res* **53**: 3193–3197

Fowlis DJ, Flanders KC, Duffie E, Balmain A and Akhurst RJ (1992) Discordant transforming growth factor β1 RNA and protein localizations during chemical carcinogenesis of the skin. *Cell Growth Diff* **3**: 81–91

Friess H, Yamanaka Y, Buchler M, Ebert M, Beger HG, Gold LI and Korc M (1993) Enhanced expression of transforming growth factor β isoforms in pancreatic cancer correlates with decreased survival. *Gastroenterology* **105**: 1846–1856

Glick AB, Kulkarni AB, Tennenbaum T, Hennings H, Flanders KC, O'Reilly M, Sporn MB, Karlsson S and Yuspa SH (1993) Loss of expression of transforming growth factor β in skin and skin tumours is associated with hyperproliferation and a high risk for malignant conversion. *Proc Natl Acad Sci USA* **90**: 6076–6080

Gomella LG, Sargent ER, Wade TP, Anglard T, Linehan WM and Kasid A (1989) Expression of transforming growth factor α in normal human adult kidney and enhanced expression of transforming growth factors α and β1 in renal cell carcinoma. *Cancer Res* **49**: 6972–6975

Gorsch SM, Memoli VA, Stukel TA, Gold LI and Arrick BA (1992) Immunohistochemical staining for transforming growth factor β1 associates with disease progression in human breast cancer. *Cancer Res* **52**: 6949–6952

Hayashi N, Ito I, Yanagisawa A, Kato Y, Nakamori S, Imaoka S, Watanabe H, Ogawa M and Nakamura Y (1995) Genetic diagnosis of lymph-node metastasis in colorectal cancer. *Lancet* **345**: 1257–1259

Hirayama D, Fujimori T, Satonaka K, Nakamura T, Kitazawa S, Horio M, Maeda S and Nagasako K (1992) Immunohistochemical study of epidermal growth factor and transforming growth factor-β in the penetrating type of early gastric cancer. *Hum Pathol* **23**: 681–685

Hoosein NM, Brattain DE, Mcknight MK, Levine AE and Brattain MG (1987) Characterization of the inhibitory effects of transforming growth factor β on a human colon carcinoma cell line. *Cancer Res* **47**: 2950–2954

Ito M, Yasui W, Kyo E, Yokozaki H, Nakayama H, Ito H and Tahara E (1992) Growth inhibition of transforming growth factor β on human gastric carcinoma cells: receptor and postreceptor signaling. *Cancer Res* **52**: 295–300

Japanese Research Society for Gastric Cancer (1995) *Japanese Classification of Gastric Carcinoma*. Kanehara & Co: Tokyo

Jatzko GR, Lisborg PH, Denk H, Klimpfinger M and Stettner HM (1995) A 10-year experience with Japanese-type radical lymph node dissection for gastric cancer outside of Japan. *Cancer* **76**: 1302–1312

Jirtle RL, Haag JD, Ariazi EA and Gould MN (1993) Increased mannose 6-phosphate/insulin-like growth factor II receptor and transforming growth factor β1 levels during monoterpene-induced regression of mammary tumours. *Cancer Res* **53**: 3849–3852

Kehrl JH, Taylor A, Kim SJ and Fauci AS (1991) Transforming growth factor-β is a potent negative regulator of human lymphocytes. *Ann NY Acad Sci* **628**: 345–353

Kim SJ, Glick A, Sporn MB and Roberts AB (1989*a*) Characterization of the promoter region of the human transforming growth factor-β1 gene. *J Biol Chem* **264**: 402–408

Kim SJ, Jeang KT, Glick AB, Sporn MB and Roberts AB (1989*b*) Promoter sequence of the human transforming growth factor-β1 gene responsive to transforming growth factor-β1 autoinduction. *J Biol Chem* **264**: 7041–7045

Kimchi A, Wang XF, Weinberg RA, Cheifetz S and Massague J (1988) Absence of TGF-β receptors and growth inhibitory responses in retinoblastoma cells. *Science* **240**: 196–199

Laiho M, DeCaprio JA, Ludlow JW, Livingston DM and Massague J (1990) Growth inhibition by TGF-β linked to suppression of retinoblastoma protein phosphorylation. *Cell* **62**: 175–185

Leof EB, Proper JA, Goustin AS, Shipley GD, DiCorleto PE and Moses HL (1986) Induction of c-sis mRNA and activity similar to platelet-derived growth factor by transforming growth factor β: a proposed model for indirect mitogenesis involving autocrine activity. *Proc Natl Acad Sci USA* **83**: 2453–2457

Mackay SL, Yaswen LR, Tarnuzzer RW, Moldawer LL, Bland KI, Copeland III EM and Schultz GS (1995) Colon cancer cells that are not growth inhibited by TGF-β lack functional type I and type II TGF-β receptors. *Ann Surg* **221**: 767–776

Maehara Y, Oshiro T, Endo K, Baba H, Oda S, Ichiyoshi Y, Kohnoe S and Sugimachi K (1996) Clinical significance of occult micrometastasis in lymph nodes from patients with early gastric cancer who died of recurrence. *Surgery* **119**: 397–402

Maruyama K, Gunven P, Okabayashi K, Sasako M and Kinoshita T (1989) Lymph node metastases of gastric cancer. General pattern in 1931 patients. *Ann Surg* **210**: 596–602

Masui T, Wakefield LM, Lechner JF, LaVeck MA Sporn MB and Harris CC (1986) Type β transforming growth factor is the primary differentiation-inducing serum factor for normal human bronchial epithelial cells. *Proc Natl Acad Sci USA* **83**: 2438–2442

© Cancer Research Campaign 1998 

Miyazono K. Hellman U. Wernstedt C and Heldin CH (1988) Latent high molecular weight complex of transforming growth factor β1. Purification from human platelets and structural characterization. *J Biol Chem* **263**: 6407–6015

Morisaki T. Katano M. Ikubo A. Anan K. Nakamura M. Nakamura K. Sato H. Tanaka M and Torisu M (1996) Immunosuppressive cytokines (IL-10. TGF-β) gene expression in human gastric carcinoma tissues. *J Surg Oncol* **63**: 234–239

Moses HL. Yang EY and Pietenpol JA (1990) TGF-β stimulation and inhibition of cell proliferation: new mechanistic insights. *Cell* **63**: 245–247

Nakamura M. Katano M. Fujimoto K and Morisaki T (1997) A new prognostic strategy for gastric carcinoma: mRNA expression of tumour growth-related factors in endoscopic biopsy specimens. *Ann Surg* **226**: 35–42

Pepper MS. Belin D. Montesano R. Orci L and Vassalli JD (1990) Transforming growth factor-beta 1 modulates basic fibroblast growth factor-induced proteolytic and angiogenic properties of endothelial cells in vitro. *J Cell Biol* **111**: 743–755

Pietenpol JA. Stein RW. Moran E. Yaciuk P. Schlegel R. Lyons RM. Pittelkow MR. Munger K. Howley PM and Moses HL (1990) TGF-β1 inhibition of c-myc transcription and growth in keratinocytes is abrogated by viral transforming proteins with pRB binding domains. *Cell* **61**: 777–785

Shipley GD. Pittelkow MR. Wille JJ Jr. Scott RE and Moses HL (1986) Reversible inhibition of normal human prokeratinocyte proliferation by type β transforming growth factor-growth inhibitor in serum-free medium. *Cancer Res* **46**: 2068–2071

Tada T. Ohzeki S. Utsumi K. Takiuchi H. Muramatsu M. Li XF. Shimizu J. Fujiwara H and Hamaoka T (1991) Transforming growth factor-β-induced inhibition of T-cell function. Susceptibility difference in T cells of various phenotypes and functions and its relevance to immunosuppression in tumour-bearing state. *J Immunol* **146**: 1077–1082

Tahara E (1990) Growth factors and oncogenes in human gastrointestinal carcinomas. *J Cancer Res Clin Oncol* **116**: 121–131

Tahara E. Sumiyoshi H. Hata J. Yasui W. Taniyama K. Hayashi T. Nagae S and Sakamoto S (1986) Human epidermal growth factor in gastric carcinoma as a biologic marker of high malignancy. *Jpn J Cancer Res* **77**: 145–152

Takanami I. Imamura T. Hashizume T. Kikuchi K. Yamamoto Y and Kodaira S (1994) Transforming growth factor β1 as a prognostic factor in pulmonary adenocarcinoma. *J Clin Pathol* **47**: 1098–1100

Torre-Amione G. Beauchamp RD. Koeppen H. Park BH. Schreiber H. Moses HL and Rowley DA (1990) A highly immunogenic tumour transfected with a murine transforming growth factor type β-1 cDNA escapes immune surveillance. *Proc Natl Acad Sci USA* **87**: 1486–1490

Travers MT. Barrett-Lee PJ. Berger U. Luqmani YA. Gazet JC. Powles TJ and Coombes RC (1988) Growth factor expression in normal. benign. and malignant breast tissue. *Br Med J* **296**: 1621–1624

Tsushima H. Kawata S. Tamura S. Ito N. Shirai Y. Kiso S. Imai Y. Shimomukai H. Nomura Y. Matsuda Y and Matsuzawa Y (1996) High levels of transforming growth factor β1 in patients with colorectal cancer: association with disease progression. *Gastroenterology* **110**: 375–382

Ueki N. Nakazato H. Ohkawa T. Ikeda T. Amuro Y. Hada T and Higashino K (1992) Excessive production of transforming growth-factor β1 can play an important role in the development of tumourigenesis by its action for angiogenesis: validity of neutralizing antibodies to block tumour growth. *Biochim Biophys Acta* **1137**: 189–196

Walker RA and Dearing SJ (1992) Transforming growth factor β1 in ductal carcinoma in situ and invasive carcinomas of the breast. *Eur J Cancer* **28**: 641–644

Welch DR. Fabra A and Nakajima M (1990) Transforming growth factor β stimulates mammary adenocarcinoma cell invasion and metastatic potential. *Proc Natl Acad Sci USA* **87**: 7678–7682

Yoshida K. Yokozaki H. Niimoto M. Ito H. Ito M and Tahara E (1989) Expression of TGF-β and procollagen type I and type III in human gastric carcinomas. *Int J Cancer* **44**: 394–398

© Cancer Research Campaign 1998